# Attrition of HIV‐exposed infants from early infant diagnosis services in low‐ and middle‐income countries: a systematic review and meta‐analysis

**DOI:** 10.1002/jia2.25209

**Published:** 2018-11-22

**Authors:** James G Carlucci, Yu Liu, Halle Friedman, Brenda E Pelayo, Kimberly Robelin, Emily K Sheldon, Kate Clouse, Sten H Vermund

**Affiliations:** ^1^ Vanderbilt Institute for Global Health Vanderbilt University Medical Center Nashville TN USA; ^2^ Division of Pediatric Infectious Diseases Department of Pediatrics Vanderbilt University Medical Center Nashville TN USA; ^3^ Department of Public Health Sciences School of Medicine and Dentistry University of Rochester Rochester NY USA; ^4^ College of Charleston Charleston SC USA; ^5^ Meharry Medical College Nashville TN USA; ^6^ Yale School of Public Health Yale University New Haven CT USA

**Keywords:** HIV, infants, low‐ and middle‐income countries, attrition, retention, loss to follow‐up

## Abstract

**Introduction:**

Identification and retention of HIV‐exposed infants in early infant diagnosis (EID) services helps to ensure optimal health outcomes. This systematic review and meta‐analysis examines the magnitude of attrition from EID services in low‐ and middle‐income countries (LMICs).

**Methods:**

We performed a comprehensive database search through April 2016. We included original studies reporting retention/attrition data for HIV‐exposed infants in LMICs. Outcomes included loss to follow‐up (LTFU), death and overall attrition (LTFU + death) at time points along the continuum of EID services. At least two authors determined study eligibility, performed data extraction and made quality assessments. We used random‐effects meta‐analytic methods to aggregate effect sizes and perform meta‐regression analyses. This study adhered to PRISMA reporting guidelines.

**Results:**

We identified 3040 unique studies, of which 92 met eligibility criteria and were included in the quantitative synthesis. The included studies represent data from 110,805 HIV‐exposed infants, the majority of whom were from Africa (77%). LTFU definitions varied widely, and there was significant variability in outcomes across studies. The bulk of attrition occurred in the first six months of follow‐up, with additional losses over time. Overall, 39% of HIV‐exposed infants were no longer in care at 18 months. When restricted to non‐intervention studies, 43% were not retained at 18 months.

**Conclusions:**

These findings underscore the high attrition of HIV‐exposed infants from EID services in LMICs and the urgent need for implementation research and resources to improve retention among this vulnerable population.

## 1 Introduction

Despite enormous progress in the areas of HIV prevention, care and treatment, HIV remains a major public health burden. According to the Joint United Nations Programme on HIV/AIDS (UNAIDS), in 2016 there was an estimated 2.1 million children (less than 15 years of age) living with HIV, 160,000 children newly infected with HIV and 120,000 children who died of HIV/AIDS 1. Notwithstanding efforts to eliminate mother‐to‐child‐transmission (EMTCT) of HIV, most of these children were infected by their HIV‐positive mothers during pregnancy, childbirth or breastfeeding (vertical transmission) 2. The vast majority of these children live in low‐ and middle‐income countries (LMICs), where domestic resources to combat vertical transmission of HIV are limited 3.

Success of paediatric HIV programmes relies on the timely identification of HIV‐exposed and HIV‐positive infants and children, enrolling them in care, and retaining them in care in order to maintain their health with life‐saving antiretroviral medications (ARVs). For HIV‐exposed infants (those born to HIV‐positive mothers), these services should begin immediately after birth and should include the provision of prophylactic ARVs throughout the period of exposure, as well as interval HIV testing until an HIV diagnosis can definitively be excluded. Infants confirmed to be HIV‐positive should be started on life‐long antiretroviral therapy (ART) 4.

HIV‐exposed infants must receive these early infant diagnosis (EID) services, along with ART‐based care for their mothers. The World Health Organization (WHO) guidelines for EID state that, “all HIV‐exposed infants [should] have HIV virological testing at four to six weeks of age or at the earliest opportunity thereafter” 5. Non‐virological testing (i.e. HIV antibody testing) can be performed after cessation of breastfeeding and/or after 18 months of age when maternal antibodies are no longer detectable in the infant. Yet, in LMICs these HIV‐exposed infants are commonly lost to follow‐up (LTFU), and many die, without completing these recommended EID services.

Previous systematic reviews of attrition from HIV services in LMIC have focused on HIV‐positive adults 6, 7, 8, HIV‐positive children 9 and on the maternal side of the EMTCT continuum of care with an emphasis on the uptake of maternal and infant HIV diagnostic services 10, 11. However, for HIV‐exposed infants, one meta‐analysis was limited to an assessment of mortality among HIV‐exposed uninfected infants compared with HIV‐unexposed uninfected infants in LMICs 12. Another review of LTFU among HIV‐exposed infants was not restricted to LMICs, focused primarily on LTFU within the first three months, did not include estimates of mortality, and only included studies published up to 2012 13.

The objective of this systematic review and meta‐analysis was to comprehensively describe the magnitude of HIV‐exposed infant attrition (LTFU and death) from EID services in LMICs, characterize selected factors that may predict poor retention of HIV‐exposed infants, and identify gaps in knowledge for future studies. Given the impending target deadline for the UNAIDS 90‐90‐90 goals 14, this work represents a timely and yet underexplored view of the extent of attrition from EID services and the paediatric HIV continuum of care.

## 2 Methods

### 2.1 Search strategy and selection criteria

For this systematic review and meta‐analysis (PROSPERO Number CRD42016034180), we searched the PubMed/MEDLINE, Embase, Cochrane Library and Web of Science electronic databases through April 2016. With the assistance of an experienced clinical librarian/information scientist, we constructed a comprehensive search strategy (Table S1). We did not restrict by publication year or language. Due to the programmatic focus of the review, the search was not limited to studies focused on any specific intervention.

Observational studies, including but not limited to clinical cohort studies, case–control studies and pre‐post design studies reporting follow‐up data on retention in care and/or attrition, were included in this review. Experimental studies, including randomized controlled trials (RCTs) were also considered if they were deemed to have external validity relative to routine programme outcomes. Experimental studies offering programme‐level rather than individual interventions were considered eligible. Also, the control groups receiving standard of care in experimental studies were sometimes included if their retention/attrition data could be disaggregated, and the decision of whether or not to include these groups was based on the available data and was at the discretion of the authors reviewing the study. Only published original reports were included; reviews, opinions, editorials and methods papers were excluded.

Criteria for study inclusion were all of the following: (1) paediatric subjects aged zero to two years; (2) reported retention/attrition/LTFU data; (3) HIV exposure/infection in subjects; and, (4) LMIC study setting as defined by the World Bank 15. Studies focused on children outside the target age range, those living outside of LMICs or HIV‐unexposed/‐uninfected populations were excluded. We also excluded studies that reported attrition outcomes but did not specify the time points at which attrition occurred. Studies reporting data including but not limited to children less than two years of age were included only if the data could be disaggregated to the population of interest.

The primary outcome measure was the proportion of HIV‐exposed infants who were not retained in care at several time intervals (0 (birth) to two months, two to six months, six to twelve months and 12 to 18 months of age/follow‐up). These selected time intervals correspond to clinically meaningful points along the continuum of EID services, namely: virological HIV testing within the first two months of life, follow‐up for results of virological testing, repeat HIV testing (either virological or non‐virological) depending on whether there is ongoing exposure to HIV (i.e. breastfeeding) and definitive non‐virological HIV testing at or after 18 months of age. If a particular study contributed multiple outcomes within a single time period, we only used the outcome associated with the later time point. For example, if 10% attrition was reported at three months and 15% attrition was reported at five months, we used 15% attrition for the period of two to six months. At each of these time points, we attempted to capture the proportion of LTFU, death and overall attrition (defined as LTFU + death). When studies did not specifically indicate the source of attrition (i.e. LTFU and/or death), the available attrition data were classified as “overall attrition.” Retention in care was defined as a patient continuing to follow‐up for appointments at a facility providing HIV EID services. Patients who transferred out to receive care at another facility were considered retained in care. Various definitions for LTFU were used in different studies, and we accepted all of these.

**Figure 1:**
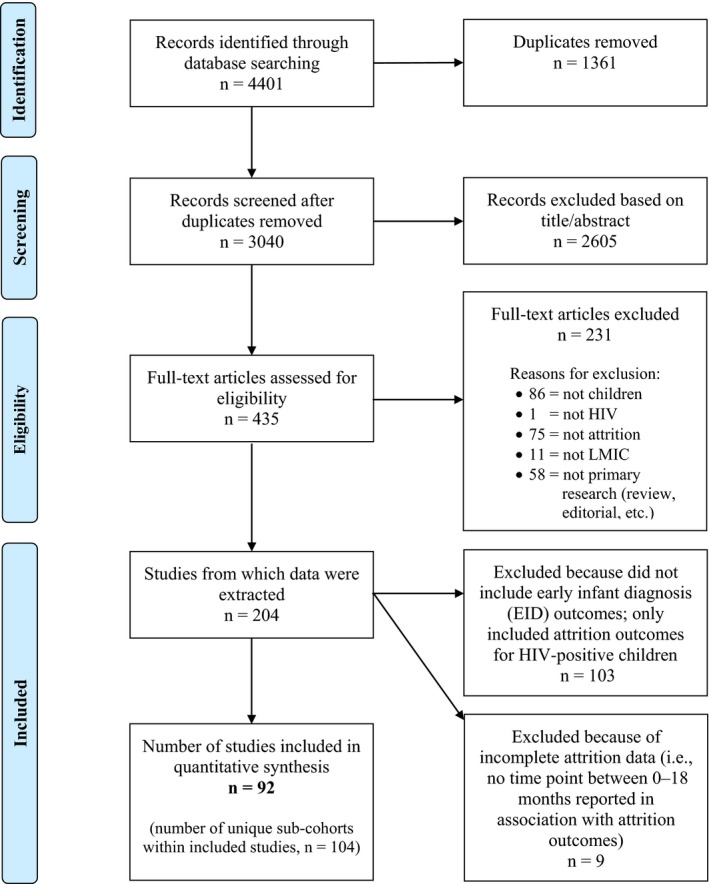
**Flow diagram for study selection.** A complete list of references included and excluded from the meta‐analysis can be found at: https://rocket.app.vumc.org/index.php?doc_id=20779.

After removing duplicate records identified in more than one of the searched databases, all unique citations and study abstracts were appraised independently for eligibility in the review by at least two study team members (JGC, HF, BEP, KR, EKS and/or SHV). For those studies meeting the inclusion criteria as detailed above, full reports for each study were obtained. These full‐text reports were also independently reviewed by at least two reviewers (JGC, HF, BEP, KR, EKS and/or SHV) for inclusion in the review. All studies not meeting the inclusion criteria were excluded. Any disagreement regarding study inclusion/exclusion, during either the screening step or the full‐text review, was arbitrated by an additional member of the study team (JGC or SHV) and resolved by consensus of the study team (Figure 1) 16.

We assessed all included studies in detail, unblinded to the names of the study investigators, their institutions, or the journals of publication. Data for each study were extracted independently by at least two study team members (JGC, HF, BEP, KR, EKS and/or SHV). All data were then cross‐checked by both of these study team members, and again any discrepancies were arbitrated by an additional member of the study team (JGC or SHV) and resolved by consensus of the study team. Extracted data were collected and managed using REDCap electronic data capture tools hosted at Vanderbilt University 17. Data elements captured included: reference details (author, publication year, journal); study details (design, inclusion/exclusion criteria, intervention if applicable, sample size, study duration, location/region of study, clinical care setting, LTFU definition); outcome details (proportions of LTFU, death, and overall attrition at specified time points, and whether case‐finding/tracing was performed to clarify outcomes for those who were not in care); and if applicable, notes on comparison populations or subcohorts within a particular study (Table S2).

The quality of studies included in the review was assessed using a standardized form, created based on the Newcastle‐Ottawa domains for assessment of cohort studies 18. The form included and scored the following items: representativeness of the population of children, adequacy of sample size, clarity of inclusion/exclusion criteria, ascertainment of HIV status, completeness of attrition/retention data, clearly defined LTFU parameters and assessments, documentation/confirmation of death, clearly documented time interval of assessment and study duration. Each item/domain was scored 0 or 1 point, and was summed to a total “quality score” that could range from zero to nine points (Table S3). Similar to our approach to study inclusion and data extraction, study quality was assessed independently by at least two reviewers (JGC, HF, BEP, KR, EKS and/or SHV), and if there was any disagreement regarding quality score, the assessment was arbitrated by an additional member of the study team (JGC or SHV) and resolved by consensus of the study team.

### 2.2 Data analysis

We employed standard meta‐analytic methods to aggregate proportions of attrition among HIV‐exposed infants in EID programmes at multiple time points of interest (0 (birth) to two months, two to six months, six to twelve months, and 12 to 18 months) and sources of attrition (LTFU, death, and overall), as defined previously. More specifically, we used the equation *Sqrt [p(1‐p)/N]* to calculate the standard error for the effect size, which was used to compute the 95% confidence interval (CI) for the effect size for the next‐step meta‐analytic aggregation. In the light of potential heterogeneity across studies, and to yield conservative estimates, we applied DerSimonian and Laird random effects analysis to generate and assign weights to each included study and to generate the pooled proportion and corresponding 95% CI. This method aims to avoid assigning greater weight to studies with larger sample sizes, and instead assigns relatively equal weights for each included study 19, 20. We used the confidence interval square test for trend to assess if the proportion of attrition varied across different time points of measurement. We used the I^2^ statistic to assess between‐study heterogeneity, with I^2^ ≤ 25% defined as low heterogeneity, I^2^ near 50% as moderate heterogeneity and I^2^ ≥ 75% as high heterogeneity 21. When applicable, a fixed‐effect model was used to adjust the pooled effect sizes when I^2^ ≤ 25%, and a random‐effects model was retained when I^2^ > 25% 21, 22.

Sensitivity/subgroup meta‐analyses were performed to determine if attrition from HIV EID services differed based on important study characteristics, including: study type (non‐intervention vs. intervention), quality score (≥5 vs. <5), study initiation year (2003 and earlier vs. 2004 and later), sample size (≥100 vs. <100), region (Asia, Americas vs. Africa) and whether case‐finding for those not in care was performed/documented (tracing vs. no tracing). The 2004 cutoff corresponds to the time period of expanded testing and antiretroviral availability in LMIC through the President's Emergency Plan for AIDS Relief (PEPFAR) and the Global Fund to Fight AIDS, Tuberculosis and Malaria (Global Fund). The sensitivity meta‐analyses stratified by whether or not tracing was performed were restricted to non‐intervention studies, since tracing efforts employed by intervention studies were likely more intensive than tracing performed in the context of routine programmatic activities.

Univariate and multivariable meta‐regression analyses were conducted to explore the potential source and magnitude of heterogeneity from *a priori* study‐ and population‐level characteristics, including: study type (intervention vs. non‐intervention), quality score (≥5 vs. <5), study initiation year (2003 and earlier vs. 2004 and later), sample size (≥100 vs. <100), hospital setting (hospital vs. non‐hospital), rurality (urban vs. rural) and region (Asia, Americas vs. Africa). Specifically, a random effects meta‐regression model based on random intercept logistic meta‐regression (binomial–normal) models was used to regress aggregate‐level data on the effect size of interest. Factors that were significant (*p *<* *0.05) in the univariate meta‐regression analyses were entered into the multivariable meta‐regression model. Adjusted R^2^ statistics generated in the meta‐regression analyses were used to quantify the magnitude of between‐study variance explained by the assessed characteristics.

We used funnel plots to graphically evaluate the presence of publication biases and Begg's and Egger's linear regression test to assess funnel plot asymmetry 23, stratified by time point of measurement and type of attrition. Stata 15.0 (StataCorp LP, College Station, Texas) was used for all statistical analyses.

This study adhered to PRISMA reporting guidelines 16. The dataset used for this meta‐analysis, a complete list of references included and excluded from the meta‐analysis, and a PRISMA checklist can be found at: https://rocket.app.vumc.org/index.php?doc_id=20779.

## 3 Results

We identified 3040 unique studies with our database search, with 92 studies deemed eligible for inclusion in this review (Figure 1 and Table S2). These 92 studies represented data from 110,805 unique HIV‐exposed infants. Among included studies, there was a median sample size of 354 participants (range: 5 to 27,174), and 80% had sample sizes ≥100 participants. The median study start year was 2007 (range: 1986 to 2013), and 70% started during or after 2004. The majority of studies were based in African countries (77%), with fewer from LMICs in Asia (16%) and the Americas (the Caribbean, Central America and South America; 7%). Most studies were conducted in urban settings (82%). The median quality score was 5 (range: 1 to 9).

Of the 92 included studies, 38% did not report a LTFU definition. Among the 62% of included studies that did report a LTFU definition there was much heterogeneity; some studies reported LTFU defined relative to the last documented encounter, some reported LTFU defined relative to missed encounters, and the intervals from last/missed encounters that qualified a person as LTFU included 60, 90, 180 days, and other time periods (Table S2).

**Figure 2:**
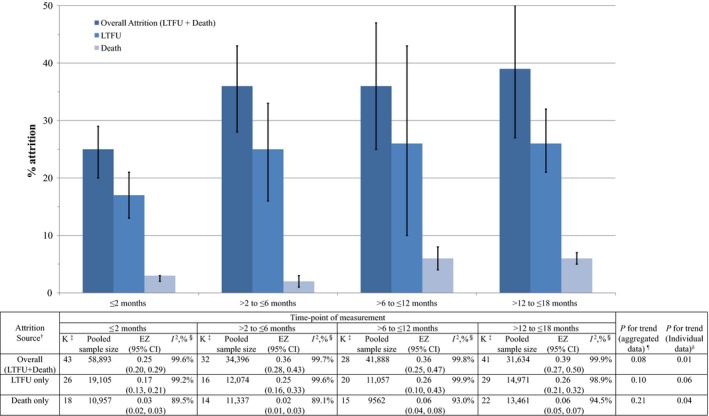
**Meta‐analysis results of attrition (loss to follow‐up (LTFU) or death) from HIV early infant diagnosis (EID) services in low‐ and middle‐income countries.** CI, confidence interval; EZ, effect size (pooled proportion); K, number of individual cohorts (may include separate effect sizes for independent samples in a single study) in the meta‐analysis; LTFU, lost to follow up. ^†^Original forest plots and meta‐analysis results are displayed in the Figures S1 to S12. ^‡^K(Death only)+K(LTFU)≤K(Overall), because cohorts included in the overall may or may not specifically indicate source of attrition (see Methods). ^§^I^2^ denotes assessment of heterogeneity of the included studies/findings, using random‐effect models to aggregate effect sizes I^2^ >25%. ^¶^Chi‐square test for trend using the pooled proportion calculated based on meta‐analysis results. ^δ^Chi‐ square test for trend using the original study‐level individual data.

Pooled and weighted meta‐analysis of the included studies showed a high prevalence of attrition from HIV EID services (Figure 2; original Forest plots shown in Figures S1 to S12). Overall attrition (LTFU + death) of HIV‐exposed infants from EID services was 25% (95% CI: 20% to 29%) by two months of age and increased to 39% (95% CI: 27% to 50%) by 18 months of age. LTFU accounted for the preponderance of overall attrition, with 17% (95% CI: 13% to 21%) of HIV‐exposed infants LTFU by two months of age and 26% (95% CI: 21% to 32%) LTFU by 18 months of age. Death accounted for a smaller proportion of overall attrition, with prevalence ranging from 3% (95% CI: 2% to 3%) by two months of age to 6% (95% CI: 5% to 7%) by 18 months of age. The proportions of paediatric patients either LTFU or dead do not sum to the proportion of overall attrition at any of the time points, because some studies contributing to the overall attrition statistics did not specifically indicate the source of attrition (i.e. LTFU or death). Therefore, it is likely that the pooled proportions of LTFU and death are underestimates of the true effect size.

While all the categories of attrition (overall, LTFU, and death) increased over time, the trend was only significant when analysing original study‐level individual data (rather than pooled proportions) for overall attrition (*p = *0.01) and death (*p *=* *0.04). However, the overall trend of attrition across time points from two months to eighteen months of age may have been skewed by the observation that the majority of LTFU and overall attrition occurred within the first six months of follow‐up and the majority of death occurred in the first 12 months of follow‐up. There was also very high heterogeneity found across age groups and attrition subgroups (I^2^ = 89.1% to 99.9%) (Figure 2 and Figures S1 to S12).

Meta‐regression analyses were performed to investigate the sources of heterogeneity (Table 1). Univariate meta‐regression analysis among infants less than two months of age showed that studies with higher quality scores (≥5) were 17.6% less likely to have overall attrition (LTFU + death; 95% CI: 5.8% to 29.3%, *p *=* *0.004) and 25.3% less likely to have attrition due to LTFU alone (95% CI: 15.8% to 34.9%, *p *<* *0.001). Similarly, multivariable meta‐regression analysis showed that studies with a higher quality score were 23.5% less likely to have attrition due to LTFU alone (95% CI: 14.1% to 32.9%, *p *<* *0.001). Univariate analyses also showed that those in intervention studies (vs. non‐intervention studies) were 13.5% less likely to have attrition due to LTFU (95% CI: 2.0% to 26.7%, *p *=* *0.047), but in the multivariable analysis, there was less statistical confidence in a true difference (7.9% decrease in attrition due to LTFU; 95% CI: 1.5% to 17.2%, *p *=* *0.094).

**Table 1:** Univariate and multivariable meta‐regression analyses of attrition from HIV early infant diagnosis services

Study characteristics	Overall (LTFU+Death)				LTFU only				Death only			
	Coefficient	95% CI	*p*‐value	% Variance explained^§^	Coefficient	95% CI	*p*‐value	% Variance explained^§^	Coefficient	95% CI	*p*‐value	% Variance explained^a^
Univariate meta‐regression (≤2 months)												
Intervention study (vs. non‐intervention)	−0.083	−0.218, 0.051	0.219	1.30	−0.135	−0.267, −0.002	0.047	11.89	0.019	−0.007, 0.045	0.142	14.31
Quality score (≥5 vs. <5)	−0.176	−0.293, −0.058	0.004	16.13	−0.253	−0.349, −0.158	<0.001	55.95	0.019	−0.011, 0.049	0.196	4.00
Study year (<2004 vs. ≥2004)	−0.071	−0.229, 0.089	0.378	−0.17	−0.011	−0.179, 0.158	0.897	−4.11	0.028	−0.003, 0.058	0.071	19.27
Sample size (≥100 vs. <100)	0.119	−0.047, 0.286	0.155	3.24	0.018	−0.153, 0.191	0.826	−3.64	−0.019	−0.059, 0.021	0.306	0.07
Non‐hospital (vs. hospital)	0.060	−0.067, 0.189	0.345	0.03	−0.034	−0.173, 0.105	0.618	−3.54	0.013	−0.014, 0.041	0.333	2.42
Rural (vs. urban)	0.119	−0.062, 0.301	0.193	2.05	−0.121	−0.331, 0.088	0.244	1.47	−0.016	−0.058, 0.026	0.442	−4.11
Region (vs. Africa)				−2.33				−0.56				−2.20
Asia	0.019	−0.151, 0.189	0.821		0.089	−0.092, 0.271	0.318		−0.015	−0.054, 0.024	0.425	
America	N/A											
Multivariable meta‐regression (≤2 months)												
Intervention study (vs. non‐intervention)					−0.079	−0.172, 0.015	0.094					
Quality score (≥5 vs. <5)					−0.235	−0.329, −0.141	<0.001					
Univariate meta‐regression (>2 and ≤6 months)												
Intervention study (vs. non‐intervention)	−0.101	−0.301, 0.101	0.314	0.11	−0.115	−0.356, 0.125	0.323	−0.59	0.001	−0.031, 0.032	0.965	−13.98
Quality score (≥5 vs. <5)	−0.103	−0.297, 0.089	0.283	0.41	−0.211	−0.465, 0.044	0.098	10.99	0.019	−0.021, 0.061	0.304	−0.24
Study year (<2004 vs. ≥2004)	−0.089	−0.319, 0.141	0.431	−0.63	0.101	−0.138, 0.339	0.381	−2.78	−0.009	−0.041, 0.021	0.494	−3.87
Sample size (≥100 vs. <100)	0.104	−0.143, 0.351	0.397	−0.05	−0.016	−0.298, 0.266	0.905	−7.28	−0.006	−0.049, 0.036	0.756	−9.38
Non‐hospital (vs. hospital)	−0.226	−0.427, −0.026	0.028	12.22	−0.185	−0.428, 0.059	0.126	9.59	0.015	−0.015, 0.046	0.297	8.42
Rural (vs. urban)	0.222	0.028, 0.415	0.026	13.19	0.221	−0.043, 0.487	0.094	15.89	0.033	0.003, 0.063	0.033	54.94
Region (vs. Africa)				7.47				−0.77				−20.01
Asia	−0.194	−0.482, 0.092	0.176		−0.089	−0.366, 0.187	0.500		−0.015	−0.061, 0.031	0.476	
America	−0.379	−0.892, 0.135	0.143		N/A				−0.014	−0.079, 0.051	0.631	
Multivariable meta‐regression (>2 and ≤6 months)												
Non‐hospital (vs. hospital)	−0.187	−0.382, 0.009	0.060									
Rural (vs. urban)	0.184	−0.005, 0.374	0.056									
Univariate meta‐regression ( 6 and ≤12 months)												
Intervention study (vs. non‐intervention)	−0.223	−0.411, −0.035	0.022	16.3	−0.087	−0.315, 0.141	0.434	−1.80	−0.014	−0.099, 0.072	0.736	−9.47
Quality score (≥5 vs. <5)	−0.163	−0.414, 0.087	0.193	3.01	−0.365	−0.648, −0.083	0.014	27.32	0.046	−0.097, 0.189	0.497	−5.08
Study year (<2004 vs. ≥2004)	−0.041	−0.074, 0.401	0.687	−3.22	0.004	−0.207, 0.216	0.965	−5.53	0.023	−0.052, 0.097	0.512	−4.51
Sample size (≥100 vs. <100)	0.163	−0.074, 0.401	0.170	3.16	0.107	−0.128, 0.342	0.353	−0.33	0.012	−0.079, 0.103	0.779	−7.84
Non‐hospital (vs. hospital)	0.026	−0.209, 0.261	0.822	−3.76	0.0002	−0.251, 0.250	0.999	−5.62	0.015	−0.063, 0.093	0.680	−7.05
Rural (vs. urban)	−0.071	−0.305, 0.162	0.535	−2.17	0.0005	−0.231, 0.232	0.996	−5.59	−0.029	−0.105, 0.047	0.428	−4.44
Region (vs. Africa)				−5.75				−9.33				−15.79
Asia	0.051	−0.214, 0.316	0.697		0.061	−0.199, 0.321	0.630		0.005	−0.078, 0.089	0.889	
America	0.112	−0.251, 0.475	0.530		−0.059	−0.545, 0.428	0.802		−0.045	−0.197, 0.106	0.529	
Multivariable meta‐regression (>6 and ≤12 months)												
N/A												
Univariate meta‐regression (>12 and ≤18 months)												
Intervention study (vs. non‐intervention)	−0.225	−0.438, −0.012	0.039	8.15	−0.171	−0.352, 0.009	0.062	8.78	0.011	−0.052, 0.727	0.730	−5.29
Quality score ≥5 (median, vs. <5)	−0.065	−0.244, 0.115	0.470	−1.18	−0.023	−0.225, 0.178	0.814	−3.79	−0.006	−0.062, 0.049	0.820	−5.36
Study year (<2004 vs. ≥2004)	−0.142	−0.309, 0.025	0.092	5.08	−0.112	−0.267, 0.042	0.148	5.17	0.028	−0.009, 0.066	0.130	6.17
Sample size (≥100 vs. <100)	0.011	−0.223, 0.245	0.927	−2.38	−0.139	−0.354, 0.077	0.199	1.20	0.015	−0.054, 0.084	0.658	−3.62
Non‐hospital (vs. hospital)	0.026	−0.156, 0.208	0.777	−2.34	−0.029	−0.198, 0.141	0.725	−3.67	−0.011	−0.049, 0.029	0.592	−3.86
Rural (vs. urban)	0.029	−0.211, 0.268	0.811	−2.45	0.065	−0.161, 0.291	0.557	−2.33	0.039	−0.011, 0.089	0.120	8.76
Region (vs. Africa)				5.56				−2.61				3.46
Asia	−0.012	−0.203, 0.179	0.898		0.073	−0.108, 0.255	0.414		−0.014	−0.062, 0.034	0.542	
America	−0.287	−0.568, −0.007	0.045		−0.125	−0.434, 0.184	0.414		−0.047	−0.108, 0.014	0.125	
Multivariable meta‐regression (>12 and ≤18 months)												
Intervention study (vs. non‐intervention)	−0.264	−0.468, −0.061	0.012		N/A							
Region (vs. Africa)	−0.338	−0.603, −0.074	0.014		N/A							

CI, confidence interval; LTFU, lost to follow‐up; N/A, univariate meta‐regression not performed due to no observations, or multivariable meta‐regression not performed due to less than two significant (*p *<* *0.05) factors in the univariate analysis.

a Between‐study variance explained reflected as adjusted R^2^ statistics.

For ages two to six months, univariate meta‐regression analysis showed a 22.6% lower likelihood of overall attrition among those cared for in a non‐hospital setting (95% CI: 2.6% to 42.7%, *p *=* *0.028), approaching statistical significance in the multivariable meta‐regression analysis (18.7% decrease in overall attrition; 95% CI: 0.9% to 38.2%, *p *=* *0.06). Univariate analysis in this age group also showed that those in rural areas were 22.2% more likely to have overall attrition (95% CI: 2.8% to 41.5%, *p *=* *0.026) and 3.3% more likely to have attrition due to death (95% CI: 3.0% to 6.3%, *p *=* *0.033), while receiving care in a rural area was of borderline significance in the multivariable analysis (increased overall attrition by 18.4%; 95% CI: 0.5% to 37.4%, *p *=* *0.056) (Table 1).

Univariate analyses among children aged six to twelve months were notable for participants in intervention studies having 22.3% lower likelihood of overall attrition (95% CI: 3.5% to 41.1%, *p *=* *0.022), and those in studies with higher quality scores (≥5) being 36.5% less likely to have attrition due to LTFU (95% CI: 8.3 to 64.8, *p *=* *0.014) (Table 1).

For children aged 12 to 18 months, both univariate and multivariable meta‐regression analyses showed significantly lower overall attrition for those in intervention studies versus non‐intervention studies (univariate: 22.5%, 95% CI: 1.2% to 43.8%, *p *=* *0.039; multivariable: 26.4%, 95% CI: 6.1% to 46.8%, *p *=* *0.012). Multivariate analysis also showed that those living in LMICs outside of Africa were 33.8% less likely to have overall attrition (95% CI: 7.4% to 60.3%, *p *=* *0.014). Study year and sample size were not significantly associated with overall attrition, LTFU alone, or death alone in univariate or multivariable analyses for any of the age groups (Table 1).

When meta‐analysis was performed using the subgroup of non‐intervention studies, even higher prevalence of attrition was observed, with overall attrition of 43% (95% CI: 30% to 56%) by 18 months. Compared with non‐intervention studies, sensitivity meta‐analysis of intervention studies had lower prevalence of LTFU and overall attrition at all time points; however, in the few intervention studies that reported death (K range: 2 to 7 studies), there was similar or slightly higher prevalence of death compared with non‐intervention studies. Studies with lower quality scores reported higher LTFU and overall attrition across all age groups, while higher attrition due to death was observed in the subgroup of studies with higher quality scores. Overall attrition appeared higher in later years (study start date ≥2004) across all age groups, but the association between study year and attrition varied by age group when examining attrition due to LTFU or death alone. The effect of sample size or region on attrition varied widely by infant age and attrition type, with no obvious trends. Sensitivity meta‐analyses stratified by studies that performed case‐finding/tracing compared to those that did not perform tracing were restricted to non‐intervention studies. By two months of age, overall attrition was 27% for both those studies that performed tracing and those that did not. Overall attrition was lower in studies that performed tracing at six months (25% vs. 41%) and 12 months (34% vs. 43%), and it was higher at 18 months (45% vs. 42%). At all time points LTFU was lower and death was higher for studies that performed tracing compared to those that did not perform tracing. There was a high degree of heterogeneity observed across studies, even when examined in sensitivity/subgroup analyses (Table 2).

**Table 2:** Sensitivity/subgroup meta‐analysis of attrition from HIV early infant diagnosis services by study characteristics

Subgroups	Time‐point of measurement (months)											
	≤2 months			>2 to ≤6 months			>6 to ≤12 months			>12 to ≤18 months		
	K	EZ (95% CI)	I^2^, %^a^	K	EZ (95% CI)	I^2^, %^a^	K	EZ (95% CI)	I^2^, %^a^	K	EZ (95% CI)	I^2^, %^a^
Overall attrition												
Study type												
Non‐intervention	29	0.27 (0.22, 0.33)	99.7	22	0.39 (0.29, 0.49)	99.8	21	0.42 (0.29, 0.54)	99.8	34	0.43 (0.30, 0.56)	99.9
Intervention	14	0.19 (0.11, 0.69)	99.4	10	0.29 (0.18, 0.39)	99.4	7	0.19 (0.10, 0.29)	99.0	7	0.20 (0.08, 0.32)	99.2
Quality score (median)												
<5	19	0.35 (0.28, 0.42)	99.2	12	0.42 (0.30, 0.55)	99.7	4	0.50 (0.09, 0.96)	99.9	14	0.43 (0.26, 0.60)	99.8
≥5	24	0.17 (0.12, 0.22)	99.6	20	0.32 (0.24, 0.40)	99.6	24	0.34 (0.24, 0.43)	99.7	27	0.37 (0.21, 0.52)	99.9
Study year												
2003 and earlier	9	0.19 (0.07, 0.30)	99.2	7	0.29 (0.10, 0.47)	99.8	7	0.33 (0.10, 0.75)	99.9	17	0.30 (0.20, 0.40)	99.2
2004 and later	34	0.26 (0.21, 0.31)	99.6	25	0.38 (0.29, 0.47)	99.7	21	0.37 (0.27, 0.47)	99.7	24	0.44 (0.29, 0.59)	99.9
Sample size												
<100	8	0.13 (0.06, 0.20)	84.6	6	0.26 (0.10, 0.42)	95.7	5	0.23 (0.13, 0.32)	65.0	7	0.37 (0.19, 0.56)	93.1
≥100	35	0.27 (0.22, 0.32)	99.7	26	0.38 (0.29, 0.46)	99.8	23	0.39 (0.26, 0.51)	99.9	34	0.39 (0.26, 0.52)	99.9
Region												
Africa	35	0.24 (0.20, 0.29)	99.6	27	0.39 (0.31, 0.48)	99.8	22	0.34 (0.22, 0.47)	99.9	26	0.42 (0.27, 0.56)	99.9
Americas	0	‐	‐	1	0.01 (0.00, 0.04)	‐	2	0.46 (0.00, 0.91)	97.1	4	0.15 (0.13, 0.17)	30.2
Asia	8	0.26 (0.13, 0.40)	99.0	4	0.14 (0.02, 0.25)	88.8	4	0.40 (0.27, 0.52)	89.7	11	0.40 (0.26, 0.55)	99.2
Case‐finding^b^												
Tracing	7	0.27 (0.07, 0.47)	99.5	4	0.25 (0.20, 0.30)	65.3	4	0.34 (0.24, 0.45)	92.5	6	0.45 (0.21, 0.69)	99.8
No tracing	23	0.27 (0.22, 0.33)	99.6	19	0.41 (0.30, 0.52)	99.8	18	0.43 (0.29, 0.57)	99.8	28	0.42 (0.28, 0.56)	99.9
LTFU only												
Study type												
Non‐intervention	17	0.23 (0.16, 0.29)	99.3	11	0.29 (0.16, 0.41)	99.7	15	0.28 (0.05, 0.51)	99.9	23	0.30 (0.24, 0.37)	98.9
Intervention	9	0.09 (0.04, 0.15)	98.7	5	0.17 (0.05, 0.29)	99.1	5	0.20 (0.08, 0.32)	98.9	6	0.13 (0.02, 0.25)	99.0
Quality score (median)												
<5	10	0.34 (0.23, 0.45)	99.2	4	0.42 (0.06, 0.79)	99.7	2	0.57 (0.16, 0.74)	99.3	6	0.28 (0.16, 0.39)	94.1
≥5	16	0.07 (0.05, 0.09)	93.1	12	0.20 (0.14, 0.25)	98.6	18	0.23 (0.17, 0.28)	97.7	23	0.26 (0.20, 0.32)	98.9
Study year												
2003 and earlier	6	0.17 (0.02, 0.33)	99.3	6	0.32 (0.11, 0.52)	99.8	7	0.27 (0.15, 0.68)	99.9	12	0.20 (0.12, 0.28)	98.6
2004 and later	20	0.18 (0.13, 0.22)	99.1	10	0.21 (0.12, 0.30)	98.8	13	0.26 (0.21, 0.32)	97.1	17	0.31 (0.24, 0.39)	99.1
Sample size												
<100	6	0.15 (0.06, 0.24)	86.0	4	0.25 (0.06, 0.44)	91.1	5	0.18 (0.06, 0.29)	80.4	5	0.39 (0.18, 0.61)	93.0
≥100	20	0.18 (0.13, 0.22)	99.4	12	0.25 (0.15, 0.34)	99.7	15	0.29 (0.10, 0.48)	99.9	24	0.25 (0.19, 0.30)	99.0
Region												
Africa	21	0.16 (0.12, 0.20)	99.2	12	0.27 (0.16, 0.37)	99.7	15	0.25 (0.06, 0.45)	99.9	19	0.26 (0.19, 0.32)	99.1
Americas	0	‐	‐	0	‐	‐	1	0.20 (0.07, 0.32)	‐	2	0.14 (0.11, 0.18)	45.4
Asia	5	0.26 (0.03, 0.49)	98.9	4	0.13 (0.02, 0.24)	88.0	4	0.31 (0.23, 0.40)	79.8	8	0.33 (0.18, 0.49)	98.5
Case‐finding^b^												
Tracing	5	0.12 (0.08, 0.17)	73.2	4	0.22 (0.19, 0.24)	0.0	4	0.27 (0.08, 0.45)	98.3	4	0.27 (0.10, 0.44)	99.5
No tracing	14	0.26 (0.18, 0.35)	99.5	8	0.30 (0.15, 0.45)	99.8	11	0.29 (0.00, 0.58)	99.9	19	0.31 (0.25, 0.37)	97.7
Death only												
Study type												
Non‐intervention	11	0.01 (0.01, 0.02)	60.6	9	0.02 (0.01, 0.03)	77.0	12	0.07 (0.04, 0.09)	93.9	20	0.06 (0.04, 0.07)	94.1
Intervention	7	0.04 (0.02, 0.06)	95.2	5	0.03 (0.01, 0.04)	94.0	3	0.05 (0.03, 0.08)	89.1	2	0.07 (0.02, 0.17)	98.0
Quality score (median)												
<5	4	0.01 (0.00, 0.02)	83.4	2	0.00 (0.00, 0.01)	0.0	1	0.02 (0.02, 0.07)	‐	3	0.07 (0.01, 0.13)	89.3
≥5	14	0.03 (0.02, 0.05)	90.7	12	0.03 (0.02, 0.03)	86.2	14	0.06 (0.05, 0.08)	93.5	19	0.06 (0.04, 0.07)	95.0
Study year												
2003 and earlier	4	0.05 (0.01, 0.09)	92.9	5	0.01 (0.00, 0.02)	84.6	5	0.08 (0.02, 0.14)	90.8	7	0.08 (0.05, 0.11)	97.6
2004 and later	14	0.02 (0.01, 0.02)	81.0	9	0.03 (0.02, 0.04)	87.9	10	0.05 (0.04, 0.07)	93.8	15	0.05 (0.04, 0.06)	87.7
Sample size												
<100	4	0.05 (0.02, 0.07)	26.1	3	0.03 (0.00, 0.06)	58.6	3	0.06 (0.01, 0.12)	80.3	2	0.05 (0.02, 0.11)	74.2
≥100	14	0.02 (0.02, 0.03)	91.5	11	0.02 (0.01, 0.03)	91.1	12	0.06 (0.05, 0.08)	94.2	20	0.06 (0.05, 0.07)	94.9
Region												
Africa	15	0.03 (0.02, 0.04)	91.3	11	0.02 (0.02, 0.03)	91.6	10	0.06 (0.04, 0.08)	94.5	16	0.07 (0.05, 0.08)	93.8
Americas	0	‐	‐	1	0.01 (0.00, 0.04)	‐	1	0.02 (0.02, 0.07)	‐	2	0.02 (0.01, 0.05)	72.5
Asia	3	0.01 (0.00, 0.01)	0.0	2	0.01 (0.00, 0.02)	0.0	4	0.07 (0.01, 0.14)	91.6	4	0.05 (0.02, 0.08)	75.4
Case‐finding^b^												
Tracing	3	0.01 (0.00, 0.03)	67.2	3	0.03 (0.00, 0.07)	85.9	3	0.10 (0.02, 0.19)	94.2	4	0.08 (0.04, 0.13)	93.6
No tracing	9	0.01 (0.00, 0.02)	61.6	7	0.01 (0.01, 0.02)	67.8	10	0.05 (0.03, 0.07)	92.4	18	0.05 (0.04, 0.06)	91.2

CI, confidence interval; EZ, pooled effect size; K, number of individual effect sizes included in the meta‐analysis; LTFU, lost to follow‐up.

a When I^2^ was ≤25%, effect size was aggregated using fixed‐effect models.

b Restricted to non‐intervention studies.

Funnel plot asymmetry was assessed using Egger's linear regression test and indicated publication bias for attrition data from infants zero to two months of age and those 12 to 18 months old, but there was no evidence of publication bias for data pertaining to infants two to twelve months of age (Table S4).

## 4 Discussion

To the best of our knowledge, this is the largest and most comprehensive assessment of HIV‐exposed infant attrition from EID services in LMICs. Our key findings include that the bulk of attrition from HIV EID services occurs during the first six months of life/follow‐up, and that by 18 months 39% of HIV‐exposed infants are no longer in care. Therefore, many HIV‐exposed infants are LTFU or die prior to receiving definitive determination of their HIV status. For some proportion of these children who were or became HIV‐positive, this attrition represents a missed opportunity for EID and initiation of potentially life‐saving ART. These findings underscore the urgent need for implementation research and resources to mitigate attrition and improve retention among this vulnerable population. By quantifying the magnitude of attrition from these programmes, these results might also serve as a benchmark for programmatic successes and weaknesses that may be useful for HIV programme directors in LMICs and global policymakers.

As expected, attrition of HIV‐exposed infants from EID services increased with duration of follow‐up. However, most of this attrition occurred by six months of age. Similarly, there is evidence that the immediate postpartum period is also a time of increased risk for attrition among HIV‐positive mothers 13, 24, 25, 26, 27. This suggests that the first six months of postpartum follow‐up might represent an especially vulnerable period, and that interventions to mitigate attrition or promote retention of mother‐infant pairs might have a greater impact if focused on and implemented early in the EID cascade. Candidate interventions might include improved integration/linkage of maternal and infant HIV services 10, 28, 29, 30, earlier and/or point‐of‐care infant virological testing 31, phone/SMS‐based messaging/reminders 29, systems engineering and continuous quality improvement approaches 32, 33, improved information systems for identifying patients who have missed clinical visits 34, 35 and enhanced community outreach and patient tracing 13, 36, 37 during this period of increased vulnerability 38.

We compared our findings to the one previous review of LTFU among HIV‐exposed infants 13, which was not restricted to LMIC, focused primarily on LTFU within the first three months, did not include estimates of mortality, and only included studies published through 2012. In this previous review, the pooled proportion of LTFU in sub‐Saharan Africa was 34%, which was similar to the overall attrition (LTFU and death) we observed in Africa by two months of age (24%) and by six months of age (39%), but higher than that observed when looking at LTFU alone in Africa by two months (16%) and by six months (27%). In terms of attrition attributable to death, our findings are similar to the rates reported in a systematic review and meta‐analysis of mortality among HIV‐exposed and HIV‐unexposed infants in LMICs 12. However, both deaths and retention are likely being underestimated 39, 40, 41; some proportion of those classified as LTFU or as overall attrition (when LTFU and/or death was not specifically reported) may have died, and some infants who “silently transferred” 42, 43, 44, 45 to other clinics and were thought to be LTFU were actually being treated elsewhere 46. Our sensitivity meta‐analysis restricted to non‐intervention studies that performed patient tracing showed lower proportions of LTFU and higher proportions of death, supporting the hypothesis that deaths and retention are being underestimated and highlighting the importance of enhanced community outreach and patient tracing. Migration of new mothers and infants during the postpartum period also has been well documented 25, 37. This presents yet another challenge: linking HIV‐exposed infants to care at a different clinic than the one to which they were referred postpartum.

There was a high degree of heterogeneity in reported outcomes across age groups and attrition subgroups. While this is also a major issue for many other meta‐analyses, this degree of between‐study variance should lead to cautious interpretation of our primary and sensitivity meta‐analyses. For example, our meta‐analysis of overall attrition by 18 months resulted in a pooled effect size of 39% (95% CI: 27% to 50%), but the effect sizes from studies contributing to the pooled estimate ranged from 3% to 99%, resulting in an I^2^ = 99.9% (Figure 2 and Figure S10). Random effects meta‐regression analyses were performed to explore the potential sources and magnitude of this heterogeneity.

While the Newcastle‐Ottawa scale has limitations, and some experts do not recommend the use of summary scores to assess the quality or risk of bias of studies included in meta‐analyses 18, 47, 48, 49, we felt that it was important for readers to have some objective measure of study quality. The Cochrane Bias Methods Group offers alternative approaches for assessing quality/bias 47, but typically these approaches are used for assessments of relatively homogeneous randomized intervention studies, rather than heterogeneous observational studies. Therefore, we elected to use a modified Newcastle‐Ottawa scale to ensure that readers are informed that the quality of data contributed by some studies may not be as reliable as the data from other studies. Along those lines, we also thought it was important to account for study quality in our meta‐regression analyses and sensitivity meta‐analyses.

Meta‐regression findings implied that higher quality scores were associated with decreased LTFU. It is possible that higher quality reports are coming from programmes providing higher quality services resulting in higher retention in care. However, it should be noted that whether death was reported and whether patient tracing was performed factored into our quality assessments (Table S3). Therefore, the association between quality score and LTFU may simply reflect more reliable reporting of death among higher quality studies. Consistent with this supposition, our sensitivity meta‐analysis restricted to non‐intervention studies that performed patient tracing showed lower proportions of LTFU and higher proportions of death.

That those cared for in non‐hospital settings (e.g. community clinics) would have less overall attrition suggests that mother‐infant pairs might be more likely to attend follow‐up in non‐hospital settings. Perhaps, this could be due to easier access to community‐based clinics (e.g. shorter travel distance, decreased wait times and less intimidating surroundings) and/or decreased perceived stigma associated with going to a community clinic versus a hospital.

Our review found that care in a rural area was associated with higher levels of attrition. This is likely due to rural areas not benefiting from the relatively higher resources and services available in more urban areas 50. Higher rural attrition is almost certainly also related to transportation, fiscal, and logistical constraints for rural mothers, fathers, and their children 51.

In sensitivity/subgroup meta‐analysis, children in intervention studies had better long‐term follow‐up. This was expected given the extra‐programmatic research resources that enhance patient follow‐up in most cases. However, death was similar in intervention and non‐intervention studies. We speculate that “real‐world” programmes (i.e. observational and non‐intervention studies) do not do as good of a job documenting death among those not retained in care as do the interventional studies. Hence, it may be that intervention studies do not have the external validity needed to generalize and compare attrition outcomes to reports of more routine programmatic data. This is also consistent with our findings that studies of lower quality reported higher overall attrition and LTFU and that studies that employed patient tracing reported lower levels of LTFU and higher levels of death. It is expected that lower quality studies would report fewer deaths since report of death was among the criteria used to determine study quality; those that did not report deaths would have received lower quality scores. Including lower quality studies may have biased our results towards higher levels of attrition. Regardless, the trend of increased attrition over time remains the same.

We stratified studies by those starting before or during/after 2004, when PEPFAR and the Global Fund began their prevention and antiretroviral treatment investments. Interestingly, attrition was higher in later years. Perhaps, in earlier years, only successful programmes were reporting outcomes (i.e. publication bias). Also, earlier publications presumably overrepresented well‐established urban programmes, while later publications represent expansion of services into newer programmes and/or rural areas 33. One would expect attrition to decline over time; increasing programmatic experience and resources from PEPFAR should result in improved retention in care and reductions in mortality. A mortality‐focused systematic review and meta‐analysis found that excess mortality among HIV‐exposed uninfected infants compared to HIV‐unexposed uninfected infants persisted after the widespread introduction of EMTCT services in LMIC, suggesting that prevention‐oriented EMTCT services alone do not necessarily reduce the risk of mortality in this population of HIV‐exposed infants 12. Together, these findings highlight the need for continued research to improve our understanding of the factors contributing to suboptimal outcomes for HIV‐exposed infants.

We were surprised to find little evidence of regional variation in attrition between Africa, Asia and the Americas, though the small number of studies from the Americas limits inference. Also, there would likely be more variability observed if we were to stratify by country rather than continent, but the number of studies from any given country was too few for us to make these comparisons.

A strength of our study was that we synthesized data from more than 110,000 HIV‐exposed infants. Consistent with the global burden of HIV and PEPFAR and Global Fund investments, most infants were from African countries, while there were relatively few studies presenting data from Asia or the Americas. Similarly, there was disproportionate representation from urban compared to rural areas. This may limit our ability to generalize our findings to settings outside of urban areas in Africa, and also highlights the need for additional outcomes research in other regions and in rural areas.

We also found that there are wide variations in the assessment and definition of LTFU, which at least in part accounts for the high degree of heterogeneity in reported outcomes across studies 52, 53. We accepted all definitions of LTFU that were provided, but because of this heterogeneity and because more than one‐third of studies did not provide a clear LTFU definition, we were not able to directly account for LTFU definition in our meta‐regression or sensitivity analyses. However, the quality assessments did consider the duration of follow‐up and whether a LTFU definition was provided, so we were at least able to partially account for the impact of LTFU definition using quality score in our meta‐regression and by performing sensitivity analysis stratified by higher versus lower quality scores. Regardless, this issue of heterogeneity in LTFU definitions and the impact of this heterogeneity on reporting of outcomes have been raised by several studies 52, 54, 55. Adoption of a universal definition for LTFU would help to standardize the reporting of attrition/retention outcomes and would allow for more reliable evaluations of comparisons between programmes.

A possible study limitation is the likelihood that some paediatric HIV publications were not represented in this analysis, possibly due to bias in peer‐reviewed publication, or due to unintentional exclusion during the database search or prior to full‐text review. However, the choice to avoid restriction by publication date or language ensured inclusivity of our search, as did our decision to include both observational and experimental studies. Our robust review criteria and inclusion/exclusion approach also increased the likelihood that the necessary studies were retained for our review and analysis.

It should also be noted that the factors/covariates used in our meta‐regression and sensitivity meta‐analyses were selected based on our experience working with HIV programmes in LMIC and on reviews of the existing literature, but surely there are other important factors that influence HIV‐exposed infant attrition from EID services (e.g. maternal health literacy). However, the studies/data utilized for our analyses were overwhelmingly routinely collected programmatic data and therefore there was not consistent exploration of factors potentially influencing these outcomes. Similarly, sex was almost always reported in aggregate (i.e. retention outcomes were rarely disaggregated by sex in the cited studies), so we were unable to assess for sex differences in EID attrition, as well. We recommend that more in‐depth exploration of the factors influencing retention in paediatric HIV services be a focus of future research.

Paediatric attrition, namely LTFU or death, is an important ongoing challenge in global HIV care and treatment programmes. Our review gives no cause for celebration, as we found no trends towards improved programme metrics over time. At the same time, metrics did not deteriorate although programmes have penetrated into remote rural areas over the past decade. Innovative programmes that have affordably and sustainably reduced child attrition should be studied for the elements of their successes that can be widely deployed 56, 57, 58.

## 5 Conclusions

This work is the largest and most comprehensive assessment of attrition of HIV‐exposed infants from EID programmes in LMICs to date. Our findings underscore the high attrition of HIV‐exposed infants from EID services in LMICs and the urgent need for implementation research and resources to mitigate attrition and improve retention among this vulnerable population. The magnitude of attrition from EID programmes in LMICs remains unacceptably high, with no significant improvements shown in combating attrition in recent years. By quantifying the magnitude of attrition from these programmes, this report might also serve as a benchmark for programmatic successes and weaknesses that may be useful for HIV programme directors in LMICs and global policymakers.

## Competing interests

The authors declare no competing interests.

## Authors’ contributions

JGC was responsible for study design, oversight, study selection, data collection, data management, preliminary data analyses, interpretation of results, creation of tables and figures, and manuscript preparation. YL was responsible for the analytic plan, statistical analyses, interpretation of results, creation of tables and figures and manuscript preparation. HF contributed to study selection, data collection, data management, creation of tables and manuscript revisions. BEP contributed to study selection, data collection and manuscript revisions. KR contributed to study selection, data collection and manuscript revisions. EKS contributed to study selection, data collection, data management and manuscript revisions. KC contributed to data management and manuscript revisions. SHV was responsible for study design, oversight, study selection, data collection and manuscript revisions.

## Supporting information


**Table S1.** Database search terms and resulting number of references
**Table S2.** Details of included studies
**Table S3.** Quality assessment tool based on the Newcastle‐Ottawa domains
**Table S4.** Results of Egger's linear regression test to assess funnel plot asymmetry for studies included in this meta‐analysis, stratified by time point and attrition type
**Figure S1.** Forest plot for overall attrition, 0 to ≤2 months.
**Figure S2.** Forest plot for attrition due to loss to follow‐up only, 0 to ≤2 months.
**Figure S3.** Forest plot for attrition due to death only, 0 to ≤2 months.
**Figure S4.** Forest plot for overall attrition, >2 to ≤6 months.
**Figure S5.** Forest plot for attrition due to loss to follow‐up only, >2 to ≤6 months.
**Figure S6.** Forest plot for attrition due to death only, >2 to ≤6 months.
**Figure S7.** Forest plot for overall attrition, >6 to ≤12 months.
**Figure S8.** Forest plot for attrition due to loss to follow‐up only, >6 to ≤12 months.
**Figure S9.** Forest plot for attrition due to death only, >6 to ≤12 months.
**Figure S10.** Forest plot for overall attrition, >12 to ≤18 months.
**Figure S11.** Forest plot for attrition due to loss to follow‐up only, >12 to ≤18 months.
**Figure S12.** Forest plot for attrition due to death only, >12 to ≤18 months.
