# Supplementary material for: Attrition of HIV‐exposed infants from early infant diagnosis services in low‐ and middle‐income countries: a systematic review and meta‐analysis
Source: J Int AIDS Soc. 2018 Nov 22;21(11):e25209. doi: 10.1002/jia2.25209 (PMC6287094; doi:10.1002/jia2.25209)
Supplement: Supplementary file 1 — Table S1. Database search terms and resulting number of references Table S2. Details of included studies Table S3. Quality assessment tool based on the Newcastle‐Ottawa domains Table S4. Results of Egger's linear regression test to assess funnel plot asymmetry for studies included in this meta‐analysis, stratified by time point and attrition type Figure S1. Forest plot for overall attrition, 0 to ≤2 months. Figure S2. Forest plot for attrition due to loss to follow‐up only, 0 to ≤2 months. Figure S3. Forest plot for attrition due to death only, 0 to ≤2 months. Figure S4. Forest plot for overall attrition, >2 to ≤6 months. Figure S5. Forest plot for attrition due to loss to follow‐up only, >2 to ≤6 months. Figure S6. Forest plot for attrition due to death only, >2 to ≤6 months. Figure S7. Forest plot for overall attrition, >6 to ≤12 months. Figure S8. Forest plot for attrition due to loss to follow‐up only, >6 to ≤12 months. Figure S9. Forest plot for attrition due to death only, >6 to ≤12 months. Figure S10. Forest plot for overall attrition, >12 to ≤18 months. Figure S11. Forest plot for attrition due to loss to follow‐up only, >12 to ≤18 months. Figure S12. Forest plot for attrition due to death only, >12 to ≤18 months. [file JIA2-21-e25209-s001.docx]

**SUPPLEMENTARY MATERIAL**

**Table S1: Database search terms and resulting number of references.**

| **Database** | **Search Terms** | **Results** |
| --- | --- | --- |
| PubMed/Medline  Original Search  Refined Search | (pediatric OR child OR children OR newborn OR infant OR neonate) AND HIV AND (stigma OR “early infant diagnosis” OR “loss to follow up” OR retention)  ("Pediatrics"[Mesh] OR pediatric [tw] OR paediatric [tw] OR "Child"[Mesh] OR child [tw] OR childhood [tw] OR children [tw] OR "Adolescent"[Mesh] OR adolescent* [tw] OR Infant [Mesh] OR infant [tw] OR "Infant, Newborn"[Mesh] OR newborn [tw] OR neonate[tw] OR baby[tw] OR babies[tw]) AND ("HIV"[Mesh] OR "HIV" [tw] OR "Human Immunodeficiency Virus" [tw] OR "acquired immunodeficiency syndrome"[MeSH Terms] OR "acquired immunodeficiency syndrome"[tw] OR "aids"[tw]) AND ("Lost to Follow-Up"[Mesh] OR "lost to follow up"[tw] OR "loss to follow up"[tw] OR "ltfu" [tw] OR "lfu"[tw] OR "attrition"[tw] OR "retention" [tw] OR "early infant diagnosis" [tw] OR “EID” [tw]) | 1218  1307 |
| Embase | (pediatric.mp. OR pediatrics/ OR paediatric.mp. OR child/ OR child.mp. OR children.mp. OR childhood.mp. OR adolescent/ OR adolescent.mp. OR adolescence.mp. OR infant/ OR infant.mp. OR newborn/ OR newborn.mp. OR neonate.mp. OR baby/ OR baby.mp. OR babies.mp.) AND (HIV.mp. OR Human immunodeficiency virus/ OR human immunodeficiency virus.mp. OR AIDS.mp. OR acquired immune deficiency syndrome/ OR acquired immune deficiency syndrome.mp.) AND (lost to follow-up.mp. OR loss to follow up.mp. OR follow up/ OR ltfu.mp. OR lfu.mp. OR retention.mp. OR attrition.mp. OR early diagnosis/ OR early infant diagnosis.mp. OR EID.mp.)  + “Exclude MEDLINE Journals” | 437 |
| Web of Science | (pediatric OR paediatric OR child OR children OR baby OR babies OR neonate OR newborn OR infant OR adolescent) AND TOPIC: (HIV OR AIDS OR "human immunodeficiency virus" OR "acquired immunodeficiency syndrome") AND TOPIC: (attrition OR retention OR "loss to follow up" OR "lost to follow up" OR "early infant diagnosis" OR EID OR "loss to follow-up" OR "lost to follow-up" OR LTFU OR LFU) Refined by: [excluding] RESEARCH AREAS: ( PHILOSOPHY OR MATHEMATICS OR MATERIALS SCIENCE OR LINGUISTICS OR FOOD SCIENCE TECHNOLOGY OR PHYSICS OR GERIATRICS GERONTOLOGY OR FISHERIES OR ENGINEERING OR BIODIVERSITY CONSERVATION OR ACOUSTICS OR ZOOLOGY OR TRANSPLANTATION OR NUCLEAR SCIENCE TECHNOLOGY OR GOVERNMENT LAW OR ANATOMY MORPHOLOGY OR AGRICULTURE ) AND [excluding] DOCUMENT TYPES: ( REVIEW OR BOOK ) Timespan: All years. Search language=Auto | 1356 |
| Cochrane Library | #1 MeSH descriptor: [Pediatrics] explode all trees  #2 MeSH descriptor: [Adolescent] explode all trees  #3 MeSH descriptor: [Child] explode all trees  #4 MeSH descriptor: [Infant] explode all trees  #5 MeSH descriptor: [Infant, Newborn] explode all trees  #6 children  #7 Neonate  #8 Paediatric  #9 baby  #10 babies  #11 #1 or #2 or #3 or #4 or #5 or #6 or #7 or #8 or #9 or #10  #12 MeSH descriptor: [HIV] explode all trees  #13 MeSH descriptor: [Acquired Immunodeficiency Syndrome] explode all trees  #14 "Human immunodeficiency virus"  #15 "AIDS"  #16 #12 or #13 or #14 or #15  #17 MeSH descriptor: [Lost to Follow-Up] explode all trees  #18 "Lost to follow up"  #19 "Loss to follow up"  #20 "Loss to follow-up"  #21 LTFU  #22 LFU  #23 Attrition  #24 Retention  #25 "Early Infant Diagnosis"  #26 EID  #27 #17 or #18 or #19 or #20 or #21 or #22 or #23 or #24 or #25 or #26  #28 #11 and #16 and #27  + subset to clinical trials | 83 |

**Table S2. Details of included studies.**

| **Source^†^**  **(First-author surname;**  **publication year)** | **Region** | **Setting**  **(rural**  **vs. urban**  **vs. both)** | **Study design** | **Follow-up**  **duration**  **(months)** | **Study**  **period** | **Analytical sample size (N)** | **Attrition type (LTFU, death, and/or overall [LTFU+ death])** | **Prevalence of attrition**  **(time point - % [n/N])** | **LTFU definition** | **LTFU tracing documented** | **Quality**  **Score** |
| --- | --- | --- | --- | --- | --- | --- | --- | --- | --- | --- | --- |
| Ahoua; 2010 | Africa | Rural | Cohort | 18 | 2000-2005 | 567 | LTFU | 18 months - 53.4% | >60 days from missed clinical visit |  | 9 |
|  |  |  |  |  |  |  | Death | 18 months - 17.4% |  | Yes |  |
|  |  |  |  |  |  |  | Overall | 18 months - 70.5% |  |  |  |
| Alcantara; 2009 | Americas | Urban | Cohort | 12 | 2000-2001 | 41 | LTFU | 12 months - 19.5% | Not reported |  | 3 |
|  |  |  |  |  |  |  | Death | 12 months - 2.4% |  | No |  |
|  |  |  |  |  |  |  | Overall | 12 months - 21.9% |  |  |  |
| Andreasson; 1993 | Africa | Urban | Cohort | 20 | 1987-1988 | 86 | LTFU | 0.25 months - 0%  12 months - 7% | Not reported |  | 6 |
|  |  |  |  |  |  |  | Death | 0.25 months - 7%  12 months - 15.1% |  | No |  |
|  |  |  |  |  |  |  | Overall | 0.25 months - 7%  12 months - 22.1% |  |  |  |
| Anoje; 2012 | Africa | Both | Cohort | 18 | 2007-2009 | 125 | LTFU | 18 months - 69.6% | Not reported |  | 5 |
|  |  |  |  |  |  |  | Death | 18 months - 7.2% |  | No |  |
|  |  |  |  |  |  |  | Overall | 18 months - 76.8% |  |  |  |
| Arreskov; 2010 | Africa | Both | Cohort | 12 | 2006-2007 | 91 | Overall | 12 months - 100% | Not reported | No | 3 |
| Azoumah; 2011 | Africa | Both | Cohort | 18 | 2004-2007 | 322 | Overall | 18 months - 8.7% | Not retained at 18 months | No | 4 |
| Badillo-Navarro; 2012 | Africa | Both | Cohort | 18 | 2008-2011 | 103 | LTFU | 18 months - 48.5% | Missing for >180 days |  | 6 |
|  |  |  |  |  |  |  | Death | 18 months - 5.8% |  | No |  |
|  |  |  |  |  |  |  | Overall | 18 months - 54.4% |  |  |  |
| Bera; 2010 | Africa | Urban | Cohort | 2 | 2006-2008 | 840 | LTFU | 2 months - 30.2% | Did not present for PCR testing by 2 months |  | 4 |
|  |  |  |  |  |  |  | Death | 2 months - 1.1% |  | No |  |
|  |  |  |  |  |  |  | Overall | 2 months - 31.3% |  |  |  |
| Bisio; 2013 | Africa | Urban | Cohort | 18 | 2005-2010 | 354 | LTFU | 18 months - 13.6% | Not retained at 18 months |  | 8 |
|  |  |  |  |  |  |  | Death | 0.1 months - 2.5%  18 months - 4.5% |  | No |  |
|  |  |  |  |  |  |  | Overall | 18 months - 18.1% |  |  |  |
| Bouraima; 2014 | Africa | Both | Cohort | 6 | 2009-2011 | 455 | Overall | 6 months - 87% | Did not receive PCR result by 6 months | No | 7 |
| Carneiro; 2001 | Americas | Both | Cohort | 3 | 1997-2000 | 70 | LTFU | 1.5 months - 0% | Not reported |  | 3 |
|  |  |  |  |  |  |  | Death | 1.5 months - 0%  3 months - 1.4% |  | No |  |
|  |  |  |  |  |  |  | Overall | 1.5 months - 0%  3 months - 1.4% |  |  |  |
| Chaisilwattana; 2002 | Asia | Both | Single-arm  efficacy trial | 18 | 1999-2000 | 109 | LTFU | From birth - 2.8%  18 months - 2.8% | Not reported | No | 5 |
|  |  |  |  |  |  |  | Overall | From birth - 2.8%  18 months - 2.8% |  |  |  |
| Chetty; 2012 | Africa | Urban | Cohort | 7 | 2008-2009 | 261 | LTFU | 0.25 months - 13%  5 months - 19.2%  6 months - 23%  7 months - 40.2% | >2 weeks from missed visit (in first 5 months), or >4 weeks from missed visit (after 5 months) |  | 8 |
|  |  |  |  |  |  |  | Death | 1 month - 0.4%  6 months - 1.5% |  | Yes |  |
|  |  |  |  |  |  |  | Overall | 0.25 months - 13%  1 month - 0.4%  5 months - 19.2%  6 months - 24.5%  7 months - 40.2% |  |  |  |
| Chi; 2007 | Africa | Urban | RCT | 1.5 | 2005-2007 | 394 | LTFU | 1.5 months - 7.6% | Not retained at 6 weeks |  | 6 |
|  |  |  |  |  |  |  | Death | 1.5 months - 2.3% |  | No |  |
|  |  |  |  |  |  |  | Overall | 1.5 months - 9.9% |  |  |  |
| Chiduo; 2013 | Africa | Both | Cohort | 2 | 2009-2011 | 4860 | LTFU | 2 months - 11.7% | Not reported | No | 4 |
|  |  |  |  |  |  |  | Overall | 2 months - 11.7% |  |  |  |
| Chokephaibulkit; 2000 | Asia | Both | RCT | 6 | 1996-1997 | 383 | LTFU | 6 months - 2.6% | Not reported |  | 7 |
|  |  |  |  |  |  |  | Death | 6 months - 1% |  | No |  |
|  |  |  |  |  |  |  | Overall | 6 months - 3.7% |  |  |  |
| Ciampa; 2011 | Africa | Rural | Cohort with  comparison (standard) | 3 | 2009-2010 | 332 | Overall | 3 months - 74.4% | No clinical visit within the first 3 months | No | 3 |
|  |  |  | (intervention) |  |  | 63 | Overall | 3 months - 46% |  |  |  |
| Ciampa; 2012 | Africa | Rural | Pre-Post  (pre-intervention) | 3 | 2009-2011 | 144 | LTFU | 3 months - 74.3% | No clinical visit within the first 3 months |  | 6 |
|  |  |  | (post-intervention #1) |  |  | 479 | LTFU | 3 months - 67.8% |  | No |  |
|  |  |  | (post-intervention #2) |  |  | 168 | LTFU | 3 months - 60.1% |  |  |  |
| Cook; 2011 | Africa | Both | Cohort | 18 | 2007-2008 | 443 | Overall | 18 months - 75% | No clinical visit within the first 18 months | No | 3 |
| Coulibaly; 2014 | Africa | Urban | Cohort | 4 | 2011-2012 | 1064 | Overall | 4 months - 70.6% | Not reported | No | 4 |
| Cruz; 2010 | Americas | Urban | Cohort | 18 | 2002-2009 | 15 | Overall | 18 months - 6.7% | Not reported | No | 1 |
| da Cruz Gouveia; 2014 | Americas | Both | Cohort | 18 | 2000-2011 | 1200 | LTFU | 18 months - 15.4% | >30 days from missed visit (first 12 months), or >60 days from missed visit (after 12 months) |  | 7 |
|  |  |  |  |  |  |  | Death | 18 months - 0.6% |  |  |  |
|  |  |  |  |  |  |  | Overall | 18 months - 16% |  | No |  |
| de Andrade; 2016 | Americas | Rural | Cohort | 18 | 1999-2011 | 1200 | Overall | 18 months - 13.6% | HIV status not determined by 18 months | No | 5 |
| Dramowski; 2011 | Africa | Urban | Cohort | 1.5 | 2007 | 147 | LTFU | 1.5 months - 66.7% | Did not receive PCR testing by 6 weeks |  | 3 |
|  |  |  |  |  |  |  | Overall | 1.5 months - 66.7% |  | No |  |
| Dravid; 2013 | Americas | Urban | Cohort | 12 | 2010-2012 | 102 | Overall | 12 months - 68.6% | Not retained at 12 months | No | 3 |
| Dube; 2012 | Africa | Both | Cohort | 2.5 | 2008-2010 | 1214 | Overall | 1.5 months - 24.2%  2.5 months - 57.1% | Did not receive PCR testing by 6 weeks or retrieve results by 10 weeks | No | 3 |
| Essomo Megnier-Mbo; 2008 | Africa | Urban | Cohort | 18 | 2002-2005 | 190 | Overall | 18 months - 68.4% | Not retained at 18 months | No | 6 |
| Feinstein; 2014 | Africa | Urban | Cohort | 18 | 2007-2008 | 335 | LTFU | 18 months - 18% | >180 days since last clinical visit |  | 8 |
|  |  |  |  |  |  |  | Death | 18 months - 8% |  |  |  |
|  |  |  |  |  |  |  | Overall | 18 months - 26% |  |  |  |
|  |  |  |  |  | 2009-2010 | 730 | LTFU | 18 months - 15% |  |  |  |
|  |  |  |  |  |  |  | Death | 18 months - 5% |  | No |  |
|  |  |  |  |  |  |  | Overall | 18 months - 20% |  |  |  |
|  |  |  |  |  | 2011-2012 | 642 | LTFU | 18 months - 18% |  |  |  |
|  |  |  |  |  |  |  | Death | 18 months - 3% |  |  |  |
|  |  |  |  |  |  |  | Overall | 18 months - 21% |  |  |  |
| Feinstein; 2015 | Africa | Urban | Cohort | 18 | 2007-2013 | 1318 | LTFU | 3 months - 9%  6 months - 13%  18 months - 18% | >180 days since last clinical visit |  | 7 |
|  |  |  |  |  |  |  | Overall | 3 months - 9%  6 months - 13%  18 months - 18% |  | Yes |  |
| Finocchario-Kessler; 2014 | Africa | Urban | Pre-Post  (pre-intervention) | 9 | 2010-2011 | 320 | Overall | 9 months - 55.9% | Not retained at 9 months | No | 6 |
|  |  |  | (post-intervention) |  | 2011-2012 | 523 | Overall | 9 months - 6.5% |  |  |  |
| Finocchario-Kessler; 2015 | Africa | Both | Cohort | 9 | 2011-2013 | 1731 | Overall | 9 months - 13.3% | Not retained at 9 months | No | 2 |
| Goodson; 2013 | Africa | Urban | Cohort | 1 | 2009-2010 | 293 | LTFU | 1 month - 13.3% | Not retained at 4 weeks |  | 5 |
|  |  |  |  |  |  |  | Overall | 1 month - 13.3% |  |  |  |
|  |  | Rural |  |  |  | 40 | LTFU | 1 month - 10% |  | Yes |  |
|  |  |  |  |  |  |  | Overall | 1 month - 10% |  |  |  |
| Goswami; 2011 | Asia | Urban | Cohort | 18 | 2004-2007 | 95 | LTFU | 18 months - 37.9% | Did not receive 18 month confirmatory testing |  | 4 |
|  |  |  |  |  |  |  | Death | 18 months - 8.4% |  |  |  |
|  |  |  |  |  |  |  | Overall | 18 months - 46.3% |  | No |  |
| Gowri; 2014 | Asia | Urban | Cohort | 18 | 2006-2011 | 56 | LTFU | 1.5 months - 25%  6 months - 28.6%  12 months - 37.5%  18 months - 46.4% | Not reported |  | 6 |
|  |  |  |  |  |  |  | Death | 1.5 months - 1.8%  6 months - 1.8%  12 months - 1.8%  18 months - 1.8% |  | Yes |  |
|  |  |  |  |  |  |  | Overall | 1.5 months - 26.8%  6 months - 30.4%  12 months - 39.3%  18 months - 48.2% |  |  |  |
| Gupta; 2016 | Asia | Both | Cohort | 1.5 | 2004-2014 | 500 | LTFU | 1.5 months - 53.4% | Not retained at 6 weeks |  | 4 |
|  |  |  |  |  |  |  | Death | 1.5 months - 0.6% |  | No |  |
|  |  |  |  |  |  |  | Overall | 1.5 months - 54% |  |  |  |
| Gupta; 2013 | Africa | Rural | Cohort | 18 | 2007-2010 | 1038 | LTFU | 18 months - 2.9% | Missed 2 consecutive visits and could not be traced |  | 9 |
|  |  |  |  |  |  |  | Death | 18 months - 7.1% |  | Yes |  |
|  |  |  |  |  |  |  | Overall | 18 months - 10% |  |  |  |
| Gupta; 2013 | Asia | Both | Cohort | 6 | 2008-2010 | 31 | LTFU | 6 months - 3.2% | Not reported |  | 3 |
|  |  |  |  |  |  |  | Death | 6 months - 0% |  | No |  |
|  |  |  |  |  |  |  | Overall | 6 months - 3.2% |  |  |  |
| Gupta; 2016 | Asia | Both | Pre-Post  (pre-intervention) | 18 | 2011-2012 | 2044 | Overall | 1.5 months - 44.1%  18 months - 54.4% | Did not receive 6 week PCR testing  Did not receive 18 month confirmatory testing |  | 4 |
|  |  |  | (post-intervention) |  | 2012-2013 | 2044 | Overall | 1.5 months - 31.6%  18 months - 45.3% |  | Yes |  |
| Hassan; 2012 | Africa | Rural | Cohort | 18 | 2006-2008 | 223 | LTFU | 2 months - 7.2%  6 months - 24.2%  12 months - 44.8%  18 months - 62.3% | Not reported |  | 6 |
|  |  |  |  |  |  |  | Death | 2 months - 0.4%  6 months - 3.1%  12 months - 4%  18 months - 4.5% |  | No |  |
|  |  |  |  |  |  |  | Overall | 2 months - 7.6%  6 months - 27.3%  12 months - 48.8%  18 months - 66.8% |  |  |  |
| Ioannidis; 1999 | Africa | Urban | RCT | 3 | 1994 | 2156 | LTFU | 3 months - 30% | Not reported |  | 4 |
|  |  |  |  |  |  |  | Death | 3 months - 0.4% |  | No |  |
|  |  |  |  |  |  |  | Overall | 3 months - 30.4% |  |  |  |
| Jackson; 2003 | Africa | Urban | RCT | 18 | 1997-2001 | 634 | LTFU | 2 months - 2.7%  18 months - 5.2% | Not reported |  | 6 |
|  |  |  |  |  |  |  | Death | 2 months - 2.2%  18 months - 12% |  | No |  |
|  |  |  |  |  |  |  | Overall | 2 months - 4.9%  18 months - 17.2% |  |  |  |
| Jamieson; 2012 | Africa | Urban | RCT | 12 | 2004-2010 | 2369 | LTFU | 0.5 months - 3.6%  7 months - 12.2%  12 months - 19.9% | Not retained at 12 months |  | 7 |
|  |  |  |  |  |  |  | Death | 7 months - 1.6%  12 months - 3.2% |  | Yes |  |
|  |  |  |  |  |  |  | Overall | 0.5 months - 3.6%  7 months - 13.8%  12 months - 23.1% |  |  |  |
| Joshi; 2010 | Asia | Both | Cohort | 18 | 2005-2008 | 305 | LTFU | 18 months - 61.3% | Not retained after tracing at 18 months |  | 6 |
|  |  |  |  |  |  |  | Death | 18 months - 3.9% |  | No |  |
|  |  |  |  |  |  |  | Overall | 18 months - 65.2% |  |  |  |
| Kebaya; 2014 | Africa | Both | RCT | 2.5 | 2013-2014 | 150 | LTFU | 2.5 months - 18% | Missed scheduled visit |  | 5 |
|  |  |  |  |  |  |  | Death | 2.5 months - 0.7% |  | No |  |
|  |  |  |  |  |  |  | Overall | 2.5 months - 18.7% |  |  |  |
| Khavari; 2014 | Africa | Urban | RCT | 60 | 1997-2003 | 963 | Death | 1.5 months - 9.2% | Not reported | No | 5 |
|  |  |  |  |  |  |  | Overall | 1.5 months - 9.2% |  |  |  |
| Kintu; 2013 | Africa | Both | RCT | 24 | 2006-2007 | 60 | LTFU | 0.5 months - 1.7% | Not reported | No | 5 |
|  |  |  |  |  |  |  | Overall | 0.5 months - 1.7% |  |  |  |
| Kuhn; 1996 | Africa | Urban | Cohort | 15 | 1996 | 234 | LTFU | From birth - 22.6%  15 months - 36.3% | Not reported |  | 4 |
|  |  |  |  |  |  |  | Death | 15 months - 10.3% |  | No |  |
|  |  |  |  |  |  |  | Overall | From birth - 22.6%  15 months - 40.6% |  |  |  |
| Kuhn; 2001 | Africa | Both | RCT | 18 | 2001 | 33 | LTFU | 18 months - 6.1% | Not reported | No | 2 |
|  |  |  |  |  |  |  | Overall | 18 months - 6.1% |  |  |  |
| Kurewa; 2012 | Africa | Urban | Cohort | 60 | 2012 | 401 | LTFU | 1.5 months - 7.5%  4 months - 21.4%  9 months - 25.2% | Missed scheduled visit and not declared dead |  | 8 |
|  |  |  |  |  |  |  | Death | 1.5 months - 2.5%  4 months - 7%  9 months - 13.2% |  | Yes |  |
|  |  |  |  |  |  |  | Overall | 1.5 months - 10%  4 months - 28.4%  9 months - 38.4% |  |  |  |
| Lilian; 2012 | Africa | Urban | Cohort | 1.5 | 2008-2010 | 838 | Overall | 1.5 months - 15.3% | Not reported | No | 4 |
| Manzi; 2005 | Africa | Rural | Cohort | 6 | 2002-2003 | 646 | LTFU | 6 months - 81.1% | >180 days since last clinical visit | No | 4 |
|  |  |  |  |  |  |  | Overall | 6 months - 81.1% |  |  |  |
| Marazzi; 2010 | Africa | Both | Cohort | 12 | 2005-2009 | 3148 | LTFU | 1 month - 1.3%  6 months - 2.9%  12 months - 11.5% | Not reported |  | 6 |
|  |  |  |  |  |  |  | Death | 1 month - 0.8%  6 months - 1.7%  12 months - 4.9% |  | No |  |
|  |  |  |  |  |  |  | Overall | 1 month - 2.1%  6 months - 4.6%  12 months - 16.4% |  |  |  |
| Martinez-Perez; 2014 | Africa | Rural | Cohort | 12 | 2011-2012 | 457 | LTFU | 12 months - 18.8% | Not reported |  | 6 |
|  |  |  |  |  |  |  | Death | 12 months - 0.9% |  | No |  |
|  |  |  |  |  |  |  | Overall | 12 months - 19.7% |  |  |  |
| Mccollum; 2011 | Africa | Urban | Pre-Post  (pre-intervention) | 12 | 2007 | 6 | LTFU | 12 months - 16.7% | >90 days since last clinical visit |  | 6 |
|  |  |  |  |  |  |  | Death | 12 months - 0% |  |  |  |
|  |  |  |  |  |  |  | Overall | 12 months - 16.7% |  | No |  |
|  |  |  | (post-intervention) |  | 2008 | 29 | LTFU | 12 months - 10.3% |  |  |  |
|  |  |  |  |  |  |  | Death | 12 months - 0% |  |  |  |
|  |  |  |  |  |  |  | Overall | 12 months - 10.3% |  |  |  |
| Merchant; 2001 | Asia | Urban | Cohort | 12 | 1996-2001 | 107 | LTFU | 12 months - 20.6% | Not reported |  | 6 |
|  |  |  |  |  |  |  | Death | 12 months - 1.9% |  | No |  |
|  |  |  |  |  |  |  | Overall | 12 months - 22.5% |  |  |  |
| Mirkuzie; 2011 | Africa | Urban | Cohort | 1.5 | 2009 | 221 | Overall | 1.5 months - 48% | Not reported | No | 4 |
| Mirkuzie; 2010 | Africa | Urban | Cohort | 18 | 2004-2009 | 8467 | Overall | 18 months - 89.4% | No HIV testing | No | 4 |
| Motswere-Chirwa; 2014 | Africa | Urban | Cohort | 2.25 | 2005-2012 | 10923 | Overall | 2.25 months-28.8% | Not reported | No | 4 |
| Mugasha; 2014 | Africa | Both | Cohort | 1.5 | 2012 | 636 | Overall | 1.5 months - 70% | Not enrolled by 6 weeks | No | 6 |
| Mwendo; 2014 | Africa | Both | Cohort | 18 | 2009-2012 | 527 | LTFU | 6 months - 49% | Not reported |  | 7 |
|  |  |  |  |  |  |  | Death | 6 months - 0.6% |  |  |  |
|  |  |  |  |  |  |  | Overall | 6 months - 49.6%  12 months - 84.1%  18 months - 98.7% |  | No |  |
| Nagot; 2016 | Africa | Both | RCT | 12.5 | 2009-2012 | 1754 | LTFU | 0.25 months-26.9%  0.75 months-28.6%  12.5 months-35.6% | Not reported |  | 4 |
|  |  |  |  |  |  |  | Death | 0.25 months - 0.6%  0.75 months - 0.6%  12.5 months - 2.5% |  | No |  |
|  |  |  |  |  |  |  | Overall | 0.25 months-27.5%  0.75 months-29.2%  12.5 months-38.1% |  |  |  |
| Namukwaya; 2015 | Africa | Both | Pre-Post  (pre-intervention) | 3.5 | 2010-2011 | 3387 | Overall | 3.5 months - 46.4% | No clinic visit by 6 weeks  No PCR testing by 14 weeks |  | 4 |
|  |  |  | (post-intervention) |  |  | 534 | LTFU | 1.5 months - 20.6% |  | No |  |
|  |  |  |  |  |  |  | Death | 1.5 months - 4.3% |  |  |  |
|  |  |  |  |  |  |  | Overall | 1.5 months - 24.9%  3.5 months - 17.4% |  |  |  |
| Nduati; 2015 | Africa | Rural | Cohort | 18 | 2006-2012 | 634 | Overall | 18 months - 73.5% | Not retained at 18 months | Yes | 6 |
| Nguefack; 2014 | Africa | Urban | Cohort | 1.5 | 2013 | 336 | Overall | 1.5 months - 47.6% | Not reported | No | 4 |
| Nlend; 2013 | Africa | Urban | Cohort | 9 | 2007-2013 | 285 | LTFU | 9 months - 14.7% | Not reported |  | 5 |
|  |  |  |  |  |  |  | Death | 9 months - 1.2% |  | No |  |
|  |  |  |  |  |  |  | Overall | 9 months - 15.9% |  |  |  |
| Nyandiko; 2010 | Africa | Both | Cohort | 18 | 2002-2007 | 2477 | LTFU | 3 months - 7.4%  18 months - 27.4% | Not reported |  | 8 |
|  |  |  |  |  |  |  | Death | 3 months - 1.3%  18 months - 3.4% |  | No |  |
|  |  |  |  |  |  |  | Overall | 3 months - 8.7%  18 months - 29.8% |  |  |  |
| Odeny; 2014 | Africa | Both | RCT | 2 | 2012-2013 | 368 | Overall | 2 months - 11.4% | Did not receive 8 week PCR testing | No | 3 |
| Okusanya; 2013 | Africa | Both | Cohort | 1.5 | 2010-2011 | 64 | LTFU | 1.5 months - 25% | Did not present for PCR testing by 6 weeks |  | 3 |
|  |  |  |  |  |  |  | Overall | 1.5 months - 25% |  | No |  |
| Oladokun; 2010 | Africa | Urban | Cohort | 18 | 2002-2007 | 303 | LTFU | 18 months - 20.8% | Did not receive 18 month confirmatory testing |  | 6 |
|  |  |  |  |  |  |  | Death | 18 months - 10.9% |  | No |  |
|  |  |  |  |  |  |  | Overall | 18 months - 31.7% |  |  |  |
| Ong'ech; 2012 | Africa | Urban | Cohort with  Comparison  (model #1) | 12 | 2008-2010 | 179 | Overall | 3.5 months - 11.7%  6 months - 19.6%  9 months - 30.2%  12 months - 37.4% | Not reported | No | 5 |
|  |  |  | (model #2) |  |  | 184 | Overall | 3.5 months - 22.8%  6 months - 43.5%  9 months - 64.1%  12 months - 51.6% |  |  |  |
| Pinchun; 1994 | Asia | Urban | Cohort | 18 | 1990-1993 | 50 | Overall | 18 months - 50% | Did not receive 18 month confirmatory testing | No | 2 |
| Poon; 2008 | Asia | Urban | Cohort | 18 | 2002-2006 | 5 | LTFU | 1.5 months - 40%  6 months - 60%  18 months - 60% | Not reported |  | 2 |
|  |  |  |  |  |  |  | Death | 18 months - 0% |  | No |  |
|  |  |  |  |  |  |  | Overall | 1.5 months - 40%  6 months - 60%  18 months - 60% |  |  |  |
| Rawizza; 2015 | Africa | Both | Cohort | 12 | 2004-2014 | 27174 | Overall | From birth - 16%  12 months - 53.3% | Not reported | No | 5 |
| Ryder; 1994 | Africa | Urban | Cohort | 36 | 1986-1990 | 333 | LTFU | 12 months - 4.8% | Not retained despite attempts at case finding |  | 8 |
|  |  |  |  |  |  |  | Death | 12 months - 16.2% |  | Yes |  |
|  |  |  |  |  |  |  | Overall | 12 months - 21% |  |  |  |
| Sagay; 2015 | Africa | Urban | Cohort | 18 | 2010-2012 | 996 | LTFU | 18 months - 14.1% | Not reported |  | 7 |
|  |  |  |  |  |  |  | Death | 18 months - 1.2% |  | No |  |
|  |  |  |  |  |  |  | Overall | 18 months - 15.3% |  |  |  |
| Sangho; 2013 | Africa | Urban | Cohort | 18 | 2005-2008 | 161 | LTFU | 18 months - 18% | Not retained at 18 months |  | 8 |
|  |  |  |  |  |  |  | Death | 18 months - 9.3% |  | No |  |
|  |  |  |  |  |  |  | Overall | 18 months - 27.3% |  |  |  |
| Scott; 2013 | Africa | Both | Cohort | 6 | 2011 | 99 | LTFU | From birth - 12%  6 months - 30% | >60 days from clinical visit |  | 7 |
|  |  |  |  |  |  |  | Death | From birth - 7%  6 months - 8% |  | No |  |
|  |  |  |  |  |  |  | Overall | From birth - 19%  6 months - 38% |  |  |  |
| Seth; 2012 | Asia | Both | Cohort | 12 | 2006-2010 | 162 | LTFU | 12 months - 29% | Not reported |  | 7 |
|  |  |  |  |  |  |  | Death | 12 months - 21% |  | No |  |
|  |  |  |  |  |  |  | Overall | 12 months - 50% |  |  |  |
| Sherman; 2004 | Africa | Both | Cohort | 12 | 2001-2002 | 1234 | LTFU | From birth - 36%  0.5 months - 44%  12 months - 94.6% | Missed clinical visit and did not return before study closure | No | 4 |
|  |  |  |  |  |  |  | Overall | From birth - 36%  0.5 months - 44%  12 months - 94.6% |  |  |  |
| Shetty; 2008 | Africa | Urban | Cohort | 18 | 2002-2004 | 727 | Overall | 1.5 months - 45.5%  18 months - 71.3% | No clinical visit by 6 weeks  <3 clinical visits by 18 months | No | 4 |
| Sidze; 2015 | Africa | Both | Cohort | 6 | 2007-2010 | 1964 | Overall | 6 months - 9.8% | >180 days from clinical visit | No | 6 |
| Singh; 2009 | Asia | Urban | Cohort | 18 | 2006-2008 | 50 | LTFU | 18 months - 56% | Not reported |  | 5 |
|  |  |  |  |  |  |  | Death | 0.25 months - 4% |  | No |  |
|  |  |  |  |  |  |  | Overall | 0.25 months - 4%  18 months - 56% |  |  |  |
| Smith; 2014 | Africa | Rural | Cohort | 2 | 2012 | 843 | Overall | 2 months - 46% | Did not receive PCR testing by 8 weeks | No | 4 |
| Sovannarith; 2012 | Asia | Urban | Cohort | 7.5 | 2008-2011 | 256 | LTFU | 1.5 months - 14.3%  7.5 months - 38.8% | Not reported |  | 5 |
|  |  |  |  |  |  |  | Death | 7.5 months - 7.4% |  | No |  |
|  |  |  |  |  |  |  | Overall | 1.5 months - 14.3%  7.5 months - 46.2% |  |  |  |
| Steel-Duncan; 2004 | Americas | Urban | Cohort | 18 | 2002-2003 | 132 | LTFU | 18 months - 11.4% | Multiple missed visits and unable to trace |  | 5 |
|  |  |  |  |  |  |  | Death | 18 months - 3.8% |  | No |  |
|  |  |  |  |  |  |  | Overall | 18 months - 15.2% |  |  |  |
| Thaithumyanon; 2001 | Asia | Urban | Cohort | 18 | 1997-2000 | 100 | LTFU | 18 months - 13% | No follow-up after leaving the nursery |  | 4 |
|  |  |  |  |  |  |  | Death | 18 months - 0% |  | No |  |
|  |  |  |  |  |  |  | Overall | 18 months - 13% |  |  |  |
| Thakwalakwa; 2014 | Africa | Rural | RCT | 18 | 2001-2012 | 248 | LTFU | 12 months - 2.4%  15 months - 4.8%  18 months - 12.1% | Not reported |  | 6 |
|  |  |  |  |  |  |  | Death | 12 months - 0%  15 months - 0%  18 months - 0% |  | No |  |
|  |  |  |  |  |  |  | Overall | 12 months - 2.4%  15 months - 4.8%  18 months - 12.1% |  |  |  |
| Thistle; 2004 | Africa | Rural | RCT | 12 | 1999-2000 | 193 | LTFU | 1.5 months - 4.1%  3 months - 9.8%  6 months - 21.8%  12 months - 36.3% | Not reported |  | 5 |
|  |  |  |  |  |  |  | Death | 1.5 months - 3.1%  3 months - 5.7%  6 months - 7.8%  12 months - 7.8% |  | No |  |
|  |  |  |  |  |  |  | Overall | 1.5 months - 7.2%  3 months - 15.5%  6 months - 29.6%  12 months - 44.1% |  |  |  |
| Vogt; 2015 | Africa | Rural | Pre-Post  (pre-intervention) | 2 | 2010-2013 | 1241 | Overall | From birth - 12.7%   - 1. months - 19%   1.5 months - 65.6%  2 months - 70.3% | No retained in specified steps along the EID cascade | Yes | 6 |
|  |  |  | (post-intervention) |  |  | 579 | Overall | From birth - 14.3%  0.1 months - 17.1%  1.5 months - 53%  2 months - 67.4% |  |  |  |
| Wang; 2009 | Asia | Both | Cohort | 18 | 2005-2009 | 644 | LTFU | 18 months - 6.8% | Not retained at 18 months |  | 8 |
|  |  |  |  |  |  |  | Death | 18 months - 7.8% |  | Yes |  |
|  |  |  |  |  |  |  | Overall | 18 months - 14.6% |  |  |  |
| Washington; 2015 | Africa | Rural | RCT | 9 | 2009-2011 | 1162 | LTFU | 9 months - 30% | Not retained for HIV testing despite phone calls and home visits |  | 5 |
|  |  |  |  |  |  |  | Death | 3 months - 5.2%  9 months - 6.2% |  | Yes |  |
|  |  |  |  |  |  |  | Overall | 3 months - 5.2%  9 Months - 36.2% |  |  |  |
| Yotebieng; 2016 | Africa | Urban | RCT | 1.5 | 2013-2014 | 397 | LTFU | 1.5 months - 10.3% | Not retained at 6 weeks |  | 5 |
|  |  |  |  |  |  |  | Death | 1.5 months - 7.3% |  | No |  |
|  |  |  |  |  |  |  | Overall | 1.5 months - 17.6% |  |  |  |

Note: EID, early infant diagnosis; LTFU, loss to follow-up; PCR, polymerase chain reaction (HIV RNA or DNA testing); RCT, randomized controlled trial

^†^ A complete list of included and excluded references can be found at: https://rocket.app.vumc.org/index.php?doc_id=20779.

**Table S3: Quality assessment tool based on the Newcastle-Ottawa domains.**

| **Question** | **Assessment** | **Comments** |  |
| --- | --- | --- | --- |
| **SELECTION** | | |  |
| 1. Representativeness of the HIV-exposed population of children | - Representative of the average in the community (†) - Selected group of children - No description of the derivation of the cohort |  |  |
| 2. Adequacy of sample size | - ≥100 (†) - <100 - Unclear/Not documented |  | |
| 3. Were inclusion/ exclusion criteria clearly described? | - Yes (†) - No/Unclear |  |  |
| 4. Ascertainment of HIV infection or infant exposure | - Mother confirmed HIV+ (†), for infant exposure - HIV confirmed with virologic test (PCR), if less than 18 months of age (†) - HIV confirmed with at least 2 non-virologic tests (rapid antibody tests), if older than 18 months (†) - Reliable clinical record (†) - Unclear if infection or exposure were confirmed |  |  |
| **OUTCOME** | | |  |
| 5. Attrition (death and LTFU) data | - Well described (†) - Incompletely described |  |  |
| 6. Assessment of outcome | - Active attempts at case-finding (†) - Reliable clinical records (†) - Poorly described |  | |
| 7. LTFU defined? | - Yes (†) - No/Unclear | LFTU definition: | |
| 8. Death documented? | - Yes (†) - No/Unclear |  | |
| 9. Adequacy of time period of the study | - ≥ 12 months of follow-up (†) - < 12 months of follow-up - Unclear/Not documented | Average length of follow-up: | |

Note: LTFU, loss to follow-up; PCR, polymerase chain reaction (HIV RNA or DNA testing)

† Up to one point awarded per question, for a total possible quality score ranging from 0-9.

**Table S4: Results of Egger’s linear regression test to assess funnel plot asymmetry for studies included in this meta-analysis, stratified by time point and attrition type.**

| **Attrition** | **Egger’s test** | |
| --- | --- | --- |
|  | **β** | ***P*-value** |
| ***≤2 months*** |  |  |
| Overall | 0.072 | 0.002 |
| LTFU only | 0.008 | 0.001 |
| Death only | 0.001 | 0.001 |
| ***>2 to ≤6 months*** |  |  |
| Overall | 0.089 | 0.085 |
| LTFU only | 0.155 | 0.713 |
| Death only | 0.003 | 0.385 |
| ***>6 to ≤12 months*** |  |  |
| Overall | 0.494 | 0.267 |
| LTFU only | 0.381 | 0.577 |
| Death only | 0.018 | 0.109 |
| ***>12 to ≤ 18 months*** |  |  |
| Overall | 0.808 | <0.001 |
| LTFU only | 0.039 | 0.002 |
| Death only | 0.002 | <0.001 |

Note: LTFU, loss to follow-up

P>0.05 indicates no publication bias

**Figure S1: Forest plot for overall attrition, 0 to ≤2 months.**

Note: ES, effect size; CI, confidence interval

Weights were determined using DerSimonian and Laird random effects analysis to avoid assigning too much weight to studies with larger sample sizes. Gray square icons surrounding effect sizes are visual representations of assigned weights.

**Figure S2: Forest plot for attrition due to loss to follow-up only, 0 to ≤2 months.**

Note: ES, effect size; CI, confidence interval

Weights were determined using DerSimonian and Laird random effects analysis to avoid assigning too much weight to studies with larger sample sizes. Gray square icons surrounding effect sizes are visual representations of assigned weights.

**Figure S3: Forest plot for attrition due to death only, 0 to ≤2 months.**

Note: ES, effect size; CI, confidence interval

Weights were determined using DerSimonian and Laird random effects analysis to avoid assigning too much weight to studies with larger sample sizes. Gray square icons surrounding effect sizes are visual representations of assigned weights.

**Figure S4: Forest plot for overall attrition, >2 to ≤6 months.**

Note: ES, effect size; CI, confidence interval

Weights were determined using DerSimonian and Laird random effects analysis to avoid assigning too much weight to studies with larger sample sizes. Gray square icons surrounding effect sizes are visual representations of assigned weights.

**Figure S5: Forest plot for attrition due to loss to follow-up only, >2 to ≤6 months.**

Note: ES, effect size; CI, confidence interval

Weights were determined using DerSimonian and Laird random effects analysis to avoid assigning too much weight to studies with larger sample sizes. Gray square icons surrounding effect sizes are visual representations of assigned weights.

**Figure S6: Forest plot for attrition due to death only, >2 to ≤6 months.**

Note: ES, effect size; CI, confidence interval

Weights were determined using DerSimonian and Laird random effects analysis to avoid assigning too much weight to studies with larger sample sizes. Gray square icons surrounding effect sizes are visual representations of assigned weights.

**Figure S7: Forest plot for overall attrition, >6 to ≤12 months.**

Note: ES, effect size; CI, confidence interval

Weights were determined using DerSimonian and Laird random effects analysis to avoid assigning too much weight to studies with larger sample sizes. Gray square icons surrounding effect sizes are visual representations of assigned weights.

**Figure S8: Forest plot for attrition due to loss to follow-up only, >6 to ≤12 months.**

Note: ES, effect size; CI, confidence interval

Weights were determined using DerSimonian and Laird random effects analysis to avoid assigning too much weight to studies with larger sample sizes. Gray square icons surrounding effect sizes are visual representations of assigned weights.

**Figure S9: Forest plot for attrition due to death only, >6 to ≤12 months.**

Note: ES, effect size; CI, confidence interval

Weights were determined using DerSimonian and Laird random effects analysis to avoid assigning too much weight to studies with larger sample sizes. Gray square icons surrounding effect sizes are visual representations of assigned weights.

**Figure S10: Forest plot for overall attrition, >12 to ≤18 months.**

Note: ES, effect size; CI, confidence interval

Weights were determined using DerSimonian and Laird random effects analysis to avoid assigning too much weight to studies with larger sample sizes. Gray square icons surrounding effect sizes are visual representations of assigned weights.

**Figure S11: Forest plot for attrition due to loss to follow-up only, >12 to ≤18 months.**

Note: ES, effect size; CI, confidence interval

Weights were determined using DerSimonian and Laird random effects analysis to avoid assigning too much weight to studies with larger sample sizes. Gray square icons surrounding effect sizes are visual representations of assigned weights.

**Figure S12: Forest plot for attrition due to death only, >12 to ≤18 months.**

Note: ES, effect size; CI, confidence interval

Weights were determined using DerSimonian and Laird random effects analysis to avoid assigning too much weight to studies with larger sample sizes. Gray square icons surrounding effect sizes are visual representations of assigned weights.
